# A Rare Case of Sinonasal Actinomycosis

**DOI:** 10.7759/cureus.100858

**Published:** 2026-01-05

**Authors:** Naoki Ashida, Naoki Umeda, Takeshi Tsuda, Maal Inoue, Hidenori Inohara

**Affiliations:** 1 Department of Otorhinolaryngology-Head and Neck Surgery, Osaka University Graduate School of Medicine, Osaka, JPN; 2 Department of Pathology, Osaka University Graduate School of Medicine, Osaka, JPN

**Keywords:** actinomycosis, emmm, endoscopic modified medial maxillectomy, endoscopic sinus surgery, odontogenic sinusitis, sinonasal infection

## Abstract

Actinomycosis is an infection caused by *Actinomyces* species--filamentous, gram-positive, anaerobic bacteria commonly found in the oral cavity. Although cervicofacial involvement is more frequently encountered, actinomycosis may involve the sinonasal tract and present with imaging findings that resemble neoplastic lesions. We report a 79-year-old woman who presented with a six-month history of left-sided purulent nasal discharge and nasal obstruction. Computed tomography (CT) and magnetic resonance imaging (MRI) revealed an expansile soft-tissue lesion with bone destruction in the left maxillary sinus, suggestive of odontogenic sinusitis. We performed endoscopic sinus surgery (ESS) combined with endoscopic modified medial maxillectomy (EMMM), which revealed marked granulation tissue and caseous purulent material without evidence of malignancy. Histopathological examination confirmed colonies consistent with *Actinomyces*. The patient received cefazolin (1 g/day) for two days postoperatively as standard perioperative antibiotic prophylaxis only, resulting in complete recovery without recurrence at six months. This case highlights the importance of considering *Actinomyces* infection in patients with unilateral maxillary sinus disease following dental procedures. Advances in endoscopic techniques, such as EMMM, allow complete debridement with minimal invasiveness and may reduce the need for prolonged therapeutic antibiotic therapy.

## Introduction

*Actinomyces* species are filamentous, anaerobic, gram-positive bacteria that commonly inhabit the oral cavity and are considered opportunistic pathogens [1]. Actinomycosis is a chronic infection that can involve various organs, most commonly the cervicofacial region, followed by the thoracic and abdominopelvic regions [2]. However, actinomycosis involving the paranasal sinuses is rare, accounting for only a small proportion of head and neck cases, with most reports limited to isolated case reports or small case series [3]. Sinonasal actinomycosis is typically a secondary infection, often arising from dental procedures or odontogenic inflammation, and the maxillary sinus is the most frequently affected site [4,5].

Diagnosis of sinonasal actinomycosis is challenging because its clinical and radiological features often mimic those of more common sinonasal malignant tumors, frequently presenting as an expansile soft-tissue mass with associated bony destruction [6-8]. As a result, diagnostic uncertainty and delayed diagnosis are not uncommon, and a high index of clinical suspicion with histopathological confirmation is required to distinguish actinomycosis from neoplastic processes [9,10]. Traditionally, prolonged antibiotic therapy has been recommended for actinomycosis [4,10]; however, the underlying rationale has not been explicitly stated in earlier literature. It has been hypothesized that this practice reflects the organism’s slow-growing nature, its tendency to involve dense fibrotic tissue, and the limited penetration of antibiotics into necrotic or poorly ventilated lesions. Notably, these treatment recommendations largely originated from an era when complete surgical clearance of sinonasal disease was often difficult to achieve. With recent advances in endoscopic sinus surgery, particularly techniques that allow wide exposure and thorough surgical debridement, the necessity of prolonged antibiotic therapy in selected noninvasive cases warrants reconsideration.

We report a rare case of sinonasal actinomycosis that was successfully treated with complete surgical debridement via endoscopic modified medial maxillectomy (EMMM) and limited perioperative prophylactic antibiotic use. This case highlights the importance of timely diagnosis and suggests that adequate surgical clearance using advanced endoscopic techniques may reduce the need for prolonged antibiotic treatment in carefully selected cases.

## Case presentation

**Figure 1:**
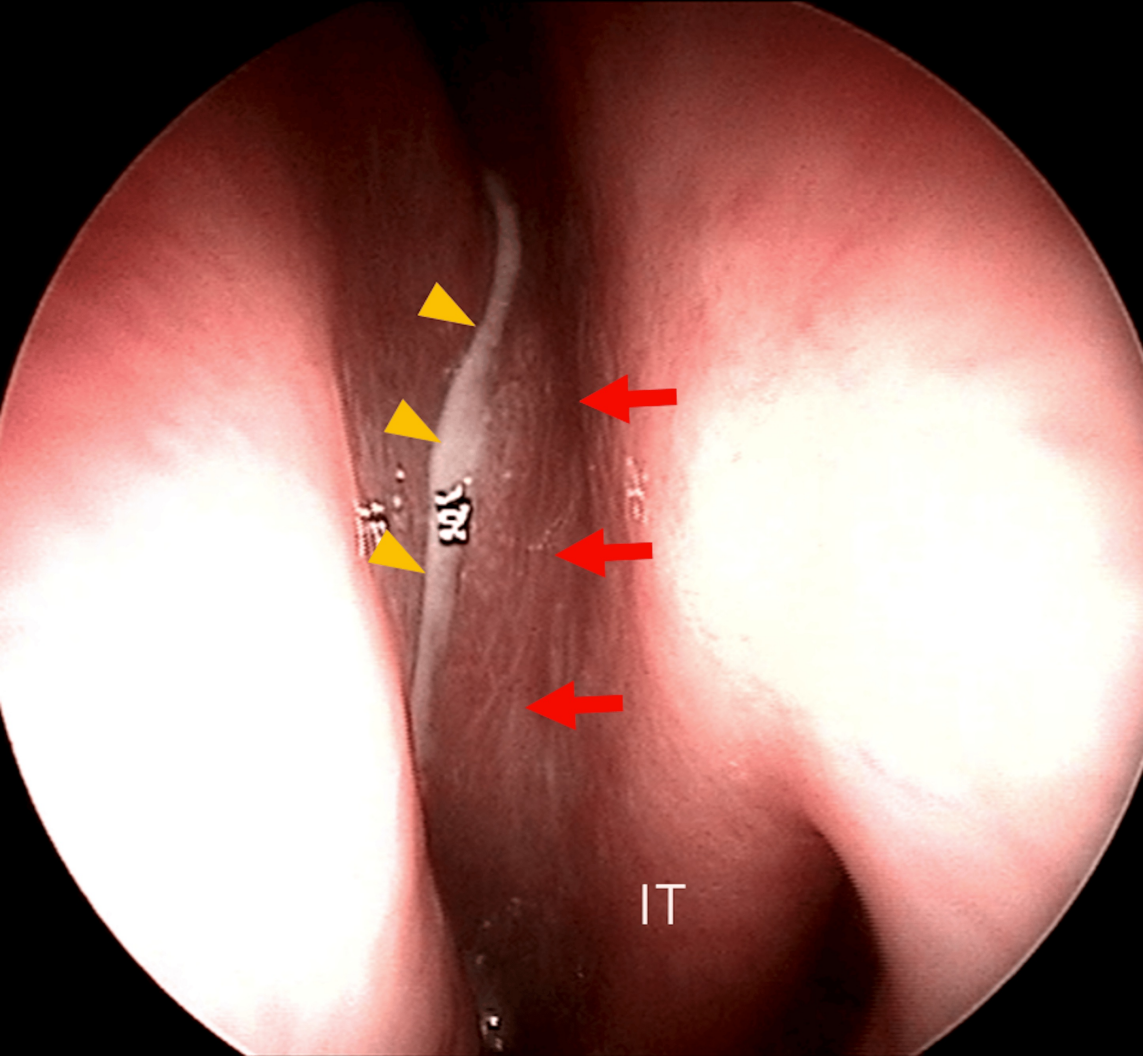
Nasal endoscopic findings Swelling of the left nasal mucosa (red arrows) and purulent discharge (yellow triangles) IT: Inferior turbinate

A 79-year-old woman presented with persistent purulent nasal discharge. She had experienced left-sided purulent rhinorrhea and nasal obstruction for six months and had been diagnosed with chronic sinusitis by a local physician. Despite treatment with oral antibiotics and expectorants, her symptoms persisted, resulting in referral to our department. Her medical history was notable for postoperative cerebral aneurysm and angina pectoris. Her dental history included a prior sinus lift with dental implant placement, followed several years later by a dental infection that required partial implant removal. She had no history of diabetes mellitus, autoimmune disease, long-term steroid use, or other conditions associated with immunodeficiency. At presentation to our hospital, endoscopic examination revealed swelling of the left lateral nasal wall with narrowing of the nasal cavity and purulent discharge from the middle meatus (Figure 1). No tumor or oroantral fistula was detected in the oral cavity.

**Figure 2:**
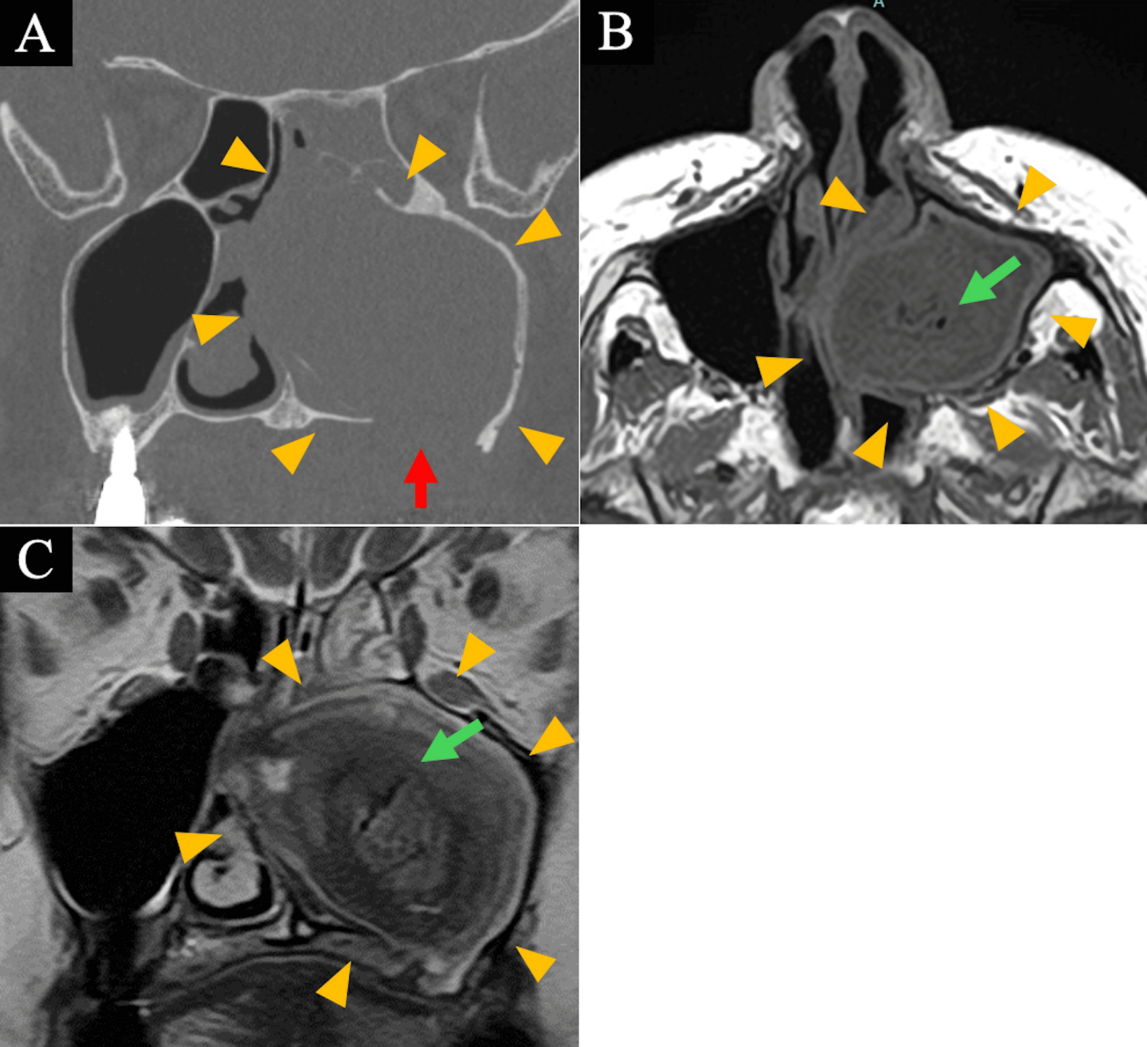
Paranasal imaging findings (A) Coronal CT scan showing an expansile soft-tissue lesion filling the left maxillary sinus with bone resorption at the sinus floor (red arrow). Yellow triangles indicate diffuse soft-tissue opacification within the left maxillary sinus. (B) Axial T1-weighted MRI demonstrating low signal intensity within the lesion. Green arrow indicates internal air retention within the soft-tissue lesion. (C) Coronal T2-weighted MRI showing iso- to low-signal intensity of the lesion, with internal air retention (green arrow).

Computed tomography (CT) showed near-complete opacification of the left maxillary sinus with expansion and bony erosion of the sinus floor and medial wall, together with rightward deviation of the nasal septum. Magnetic resonance imaging (MRI) revealed an expansile soft-tissue lesion with mixed low-to-iso intensity on T2-weighted images and low intensity on T1-weighted images, with internal air retention (Figures 2A-2C).

Given these imaging findings, odontogenic sinusitis was considered likely, while a neoplastic process remained in the differential. Therefore, surgical intervention was planned.

**Figure 3:**
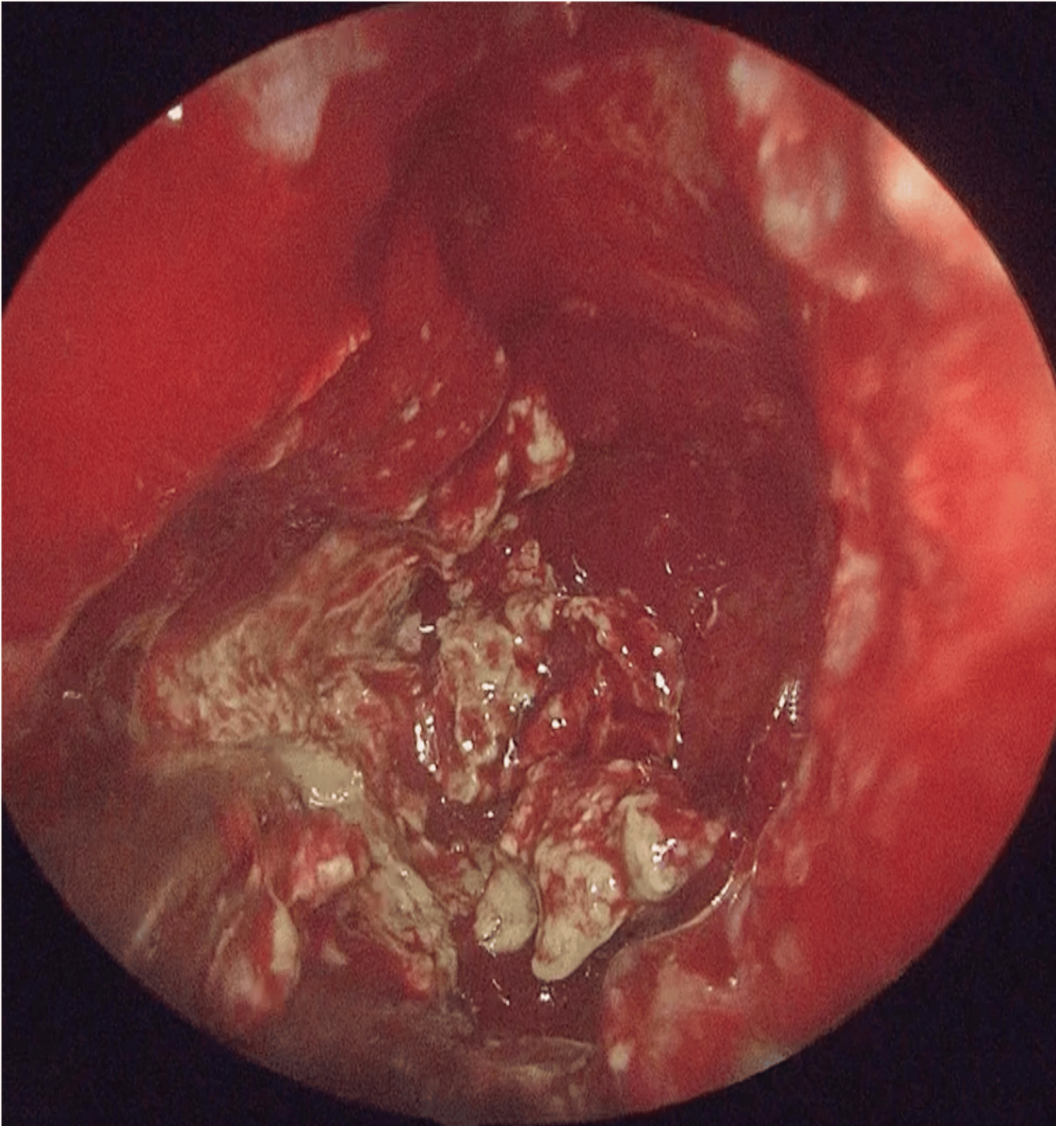
Intraoperative findings Caseous material observed within the maxillary sinus

Under general anesthesia, left endoscopic sinus surgery (ESS) and septoplasty were performed. The paranasal mucosa exhibited prominent granulation tissue, and caseous purulent discharge was present in the maxillary sinus (Figure 3).

**Figure 4:**
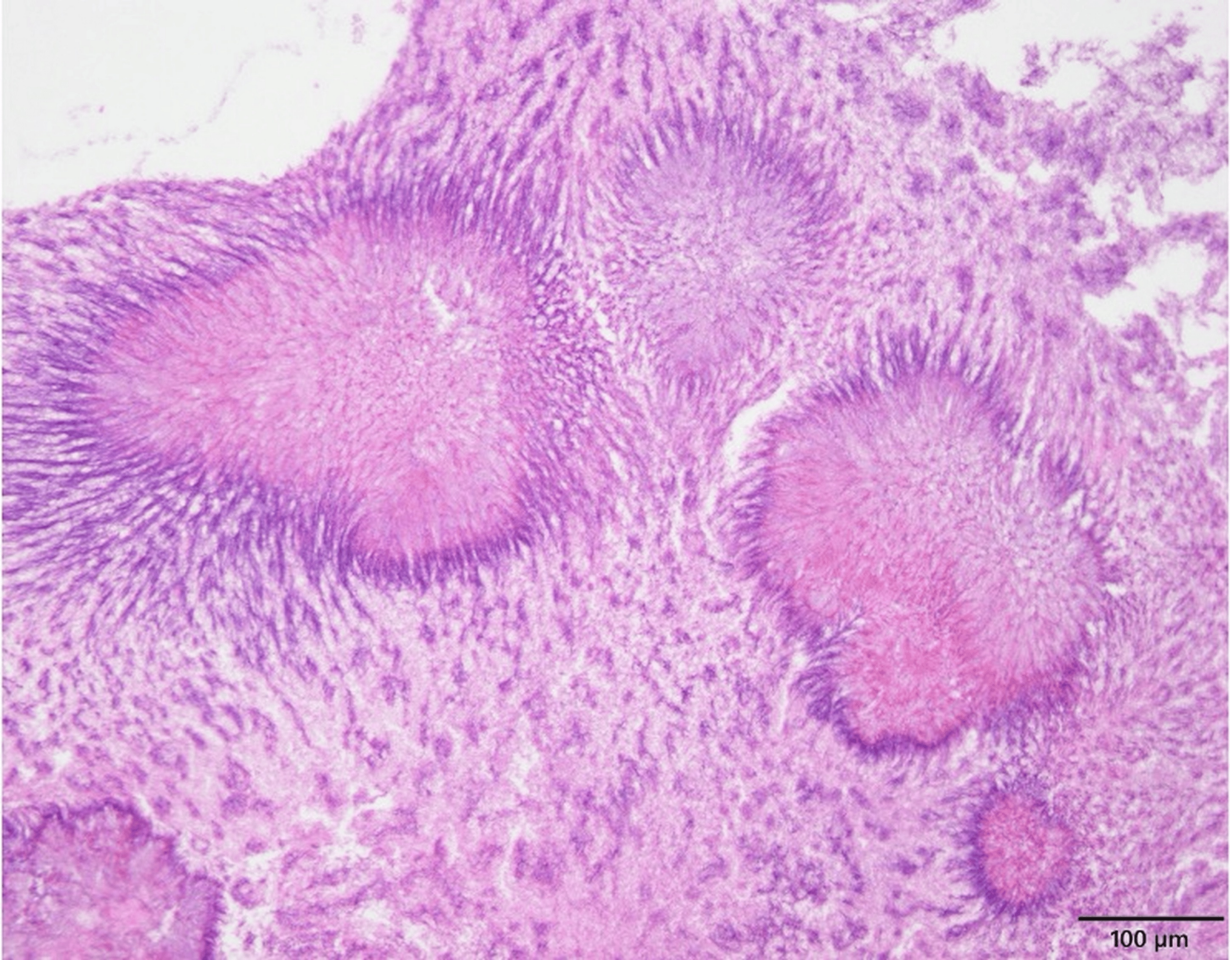
Postoperative pathological findings Basophilic filaments arranged in a radial pattern, forming a bacterial mass with associated necrosis were observed within the maxillary sinus contents, consistent with *Actinomyces* infection.

For better visualization and debridement, we proceeded with EMMM, which provides wide access to the maxillary sinus while preserving the inferior turbinate and nasolacrimal duct. Intraoperative frozen section showed no neoplastic lesion. The bone-destructive maxillary sinus floor was covered with granulation tissue, and no communication with the oral cavity was identified. The retained material was removed, and the sinus cavity was irrigated thoroughly with saline. On postoperative pathological evaluation, basophilic filaments arranged in a radial pattern, forming a bacterial mass with associated necrosis, were identified within the maxillary sinus contents, consistent with *Actinomyces* (Figure 4). Although microbiological cultures were also performed, coagulase-negative *Staphylococci *were isolated, and no *Actinomyces* species were detected.

**Figure 5:**
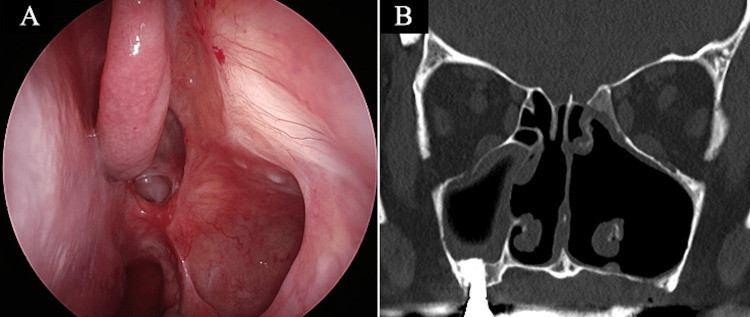
Six-month postoperative findings (A) Nasal endoscopic view; (B) CT scan demonstrating restored aeration of the maxillary sinus

The patient received cefazolin (1 g/day) for two days postoperatively as standard perioperative antibiotic prophylaxis only, with no additional therapeutic antibiotic therapy. She was discharged on postoperative day five and continued nasal irrigation. At the time, actinomycosis was histopathologically confirmed, and standard perioperative antibiotic prophylaxis had already been completed. As the patient showed favorable postoperative clinical and endoscopic findings, no additional or prolonged antibiotic therapy was administered. At six months postoperatively, the maxillary sinus remained well-aerated, and her symptoms had completely resolved without recurrence (Figures 5A-5B).

## Discussion

*Actinomyces* are filamentous, anaerobic, gram-positive rods that commonly colonize the oral cavity, gastrointestinal tract, and female genital tract, but they can become pathogenic in areas of tissue injury or inflammation [1,3,4]. Cervicofacial infections account for approximately 50-65% of all actinomycosis cases, followed by the thoracic (15-30%) and abdominopelvic (20%) regions [2]. Paranasal sinus involvement is rare [3].

Most sinonasal cases are secondary odontogenic sinusitis, arising from dental infection or penetration of dental materials into the maxillary sinus [5]. In the present case, CT demonstrated extensive bone destruction at the maxillary sinus floor, and the patient had a history of dental procedures. Thus, it is presumed that *Actinomyces* from the oral cavity may have entered the maxillary sinus through an oroantral fistula associated with odontogenic infection. The fistula likely closed spontaneously, allowing the bacteria to proliferate in an anaerobic environment, leading to worsening infection.

Although actinomycosis generally responds well to antibiotics, it can occasionally extend beyond the paranasal sinuses, resulting in orbital abscess, abducens nerve palsy, subdural abscess, or brain abscess [6,11-13]. Therefore, timely diagnosis and appropriate treatment are crucial. Radiologically, expansile unilateral maxillary sinus opacification and calcified lesions are characteristic findings, but these are nonspecific and can mimic sinonasal tumors [6-8]. For this reason, a combination of Gram staining, bacterial culture, and histopathology is important for diagnosis. Histologically, sulfur granules are pathognomonic-basophilic masses surrounded by eosinophilic terminal clubs and filamentous gram-positive rods on H&E staining [9]. Because *Actinomyces* are anaerobic, cultures are often negative [13]; hence, histopathological confirmation is essential.

The standard treatment for sinonasal actinomycosis involves a combination of surgical debridement and antibiotic therapy, as antibiotics alone are rarely curative [10]. Traditionally, prolonged antibiotic therapy has been recommended, often extending for several weeks to months [4,10]. However, recent studies have suggested that a shorter course of antibiotics may be sufficient when the disease is noninvasive and complete surgical debridement is achieved [3,12-14]. In particular, the eradication of the infectious focus and restoration of adequate sinus ventilation appear to be key determinants of successful outcomes.

With recent advances in endoscopic sinus surgery, especially minimally invasive approaches such as EMMM, wide exposure of the maxillary sinus has become possible, allowing for thorough and complete removal of lesions [15]. In the present case, complete debridement achieved through EMMM may have contributed to the favorable outcome, allowing avoidance of prolonged therapeutic antibiotic therapy and limiting antibiotic use to perioperative prophylaxis.

This case supports the concept that, in selected noninvasive cases, adequate surgical clearance may reduce the need for prolonged antibiotic treatment. Nevertheless, this report has several limitations. First, it represents a single case involving a patient without major comorbidities such as immunodeficiency. Second, the follow-up period was relatively short, limited to six months, which precludes broader generalization.

## Conclusions

This case highlights the importance of considering *Actinomyces* infection in patients with unilateral maxillary sinus disease, especially those with a history of dental procedures, as its clinical and radiological presentation can mimic malignancy. Thorough surgical debridement, facilitated by advanced techniques, such as EMMM, may play an important role in successful disease control. In selected cases, adequate surgical intervention may reduce the need for prolonged therapeutic antibiotic therapy, potentially limiting the adverse effects associated with traditionally recommended long-term treatment. Timely diagnosis and appropriate surgical management may therefore contribute to favorable outcomes in rare cases of sinonasal actinomycosis.
